# HLR-UFS: Hessian-based Unsupervised Feature Selection using Low-Rank Approximation

**DOI:** 10.1371/journal.pone.0356837

**Published:** 2026-09-03

**Authors:** Jiyan Zhang, Weihan Lin, Yanfang Liu, Shuzhen Tu, Ming Peng

**Affiliations:** 1 College of Mathematics and Information Engineering, Longyan University, Longyan, China; 2 Fujian Provincial University Key Laboratory of Big Data Mining and Applications, Longyan University, Longyan, China; Islamia University of Bahawalpur: The Islamia University of Bahawalpur Pakistan, PAKISTAN

## Abstract

Graph-based Laplacian regularization techniques have been extensively applied in unsupervised feature selection due to their capability in capturing the inherent structure of data. However, Laplacian regularization frequently results in a solution that is biased towards a constant geodesic function, which leads to inadequate extrapolation capabilities and an inability to robustly maintain the data’s topological structure. Aiming to tackle the drawback, we propose a new framework named Hessian-based Unsupervised Feature Selection using Low-Rank Approximation (HLR-UFS). First, our method introduces Hessian regularization to address the inability of graph-based Laplacian regularization to effectively preserve complex topological structures and nonlinear geometric information due to null space constraints. Second, to explicitly eliminate feature redundancy, we use low-rank approximation techniques to identify latent correlations among features. Furthermore, we employ $\ell_{2,1}$-norm regularization to suppress noise and outliers. Finally, we design an efficient algorithm and rigorously validate its convergence through theoretical analysis. Comprehensive assessments on nine standard datasets show the validity and superior performance of HLR-UFS.

## 1. Introduction

Rapid development in the field of computer technology has led to the emergence of high-dimensional data in many practical application areas, including image processing, video surveillance systems, speech data, and biological information processing [1–3]. This has made dimensionality reduction an increasingly important yet challenging problem in machine learning, computer vision, and pattern recognition applications [4,5]. In practice, dimensionality reduction techniques can be broadly categorized into two main paradigms: feature selection, which identifies optimal feature subsets, and feature extraction, which constructs new feature representations. Feature extraction techniques, including manifold learning [6] and principal component analysis (PCA) [7], transform data from a high-dimensional to a low-dimensional space, which may reduce the interpretability of the original features. In contrast, feature selection specifically identifies and retains key subsets from the original feature set, safeguarding the vital information inherent in the full-dimensional space [8–10]. Consequently, feature selection has become a crucial technique in high-dimensional data analysis, offering a balance between dimensionality reduction and the preservation of semantic meaning.

Feature selection techniques are typically categorized into two types, contingent upon the availability of class labels: supervised and unsupervised. The supervised approaches leverage explicit label annotations to identify maximally discriminative feature subsets, benefiting from well-defined guidance that enables efficient optimization for specific learning objectives [11–13]. Nevertheless, supervised methods face fundamental implementation challenges, as large-scale data annotation typically incurs substantial time and resource costs, especially in domains requiring specialized expertise. The development of methods for unsupervised feature selection is propelled by this inherent constraint. Unsupervised feature selection techniques eliminate reliance on labeled data by leveraging intrinsic data properties, such as manifold structures or cluster relationships, to construct pseudo-supervisory signals for feature selection. Although unsupervised feature selection holds significant theoretical promise, its practical effectiveness critically depends on the quality of pseudo-supervision derived from learned structural patterns. Therefore, accurately capturing data structures remains an important and ongoing research frontier in machine learning.

Intrinsic data structures are often used as pseudo-supervisory signals in unsupervised feature selection. Thus, structure learning is frequently employed in unsupervised feature selection, and a particularly efficient method is graph-based regularization. It relies on the standard assumption that data points exhibiting similarity in the original data space should still demonstrate similarity in the transformed space [14,15]. From this principle arises the widely recognized graph-based Laplacian regularization term, critical for depicting the data’s intrinsic structure [16]. Despite its widespread use in feature selection, the graph-based Laplacian regularization faces certain limitations when it comes to geodesic and null space considerations. Furthermore, graph-based Laplacian regularization primarily captures pairwise linear relationships between samples and fails to model high-order geometric properties such as manifold curvature or topological connectivity [17].

In addition, the problem of feature selection can be conceptualized as seeking out the subset of features that optimally mirrors the initial feature space in a lower-dimensional subspace, which is fundamentally consistent with the low-rank approximation approach [18,19]. Low-rank approximation constitutes an effective structural prior that promotes compact latent dependency patterns while mitigating feature redundancy. Existing low-rank-based unsupervised feature selection methods usually exploit the intrinsic low-dimensional structure of data through the low-rank constraint [20], Schatten-*p* norm regularization [21], or nuclear-norm regularization [22]. In this work, low-rank approximation is employed not as a strict assumption on the raw input data matrix, but as a regularization principle to induce a compact and informative feature-selection structure.

Recent research in unsupervised feature selection has also extended toward deep representation learning, where autoencoder-based architectures jointly incorporate manifold preservation and feature selection within a unified training framework [23]. These methods are effective in modeling nonlinear latent structures, but they typically optimize representation learning and feature selection simultaneously. In this work, we instead focus on an interpretable, optimization-based formulation.

Against this backdrop, we aim to address the limitations of existing graph-based regularization while integrating the advantages of low-rank approximation. To this end, we propose a novel method termed Hessian-based Unsupervised Feature Selection using Low-Rank Approximation (HLR-UFS). First, we harness Hessian regularization to capture the local geometric structure of complex nonlinearities, consequently effectively preserving the data’s topological structure. Second, building upon self-representation principles, we impose a low-rank prior on the coefficient matrix to capture a compact global dependency structure among features, while $\ell_{2,1}$-norm regularization is employed to improve robustness to noise and outliers. Finally, we compare our novel algorithm with both the baseline and several leading-edge feature selection methods using nine datasets that are openly available from a machine learning database. Broad-ranging experiments confirm that our method achieves superior clustering performance relative to other methods across the tested datasets. In summary, the primary contributions of this paper are highlighted as follows:

- We replace traditional Laplacian regularization with Hessian regularization to capture high-order geometric structures, overcoming the linearity limitation of traditional graph-based methods.
- We impose low-rank approximation on the coefficient matrix to capture shared latent structure among features and promote a compact feature-selection structure, while $\ell_{2,1}$-norm regularization enhances robustness to noise and outliers.
- We validate HLR-UFS extensively on nine datasets spanning images, biology, and mass spectrometry. Extensive experiments convincingly demonstrate the superiority of HLR-UFS.

This paper is structured as follows: Sect 2 provides a comprehensive review of existing methods. Sect 3 introduces the proposed HLR-UFS framework. Sect 4 presents the IRLS-based optimization algorithm, algorithmic procedure, and computational complexity analysis of HLR-UFS. Sect 5 analyzes the algorithm’s optimization convergence. Sect 6 displays and analyzes the results of experiments. Finally, Sect 7 concludes with an overview of the paper and future research directions.

## 2. Related work

Over the past few decades, a variety of unsupervised feature selection techniques have been proposed to address high-dimensional data when label information is unavailable or limited. Zhu et al. employ the self-representation property and $\ell_{2,1}$-norm regularization to effectively identify representative features [24]. Tang et al. integrate latent representation learning within the feature selection process to leverage the interrelations among data and conduct feature selection in the latent space, thereby enhancing noise resilience [25]. Ma et al. incorporate a latent relationship penalty term into unsupervised feature selection to account for sample uniqueness, using an attribute score strategy to mitigate noise interference and preserve data structure [26]. Liu et al. employ $\ell_{2,p}$-norm feature reconstruction with an adaptive distance metric, graph learning, and inner product regularization to enhance robustness, preserve data structure, and ensure feature sparsity [27].

A primary concern of unsupervised feature selection is maintaining the inherent local geometric relationships within the dataset [28]. Therefore, the graph-based Laplacian regularization term is frequently adopted in embedded unsupervised feature selection methods. He et al. propose the Laplacian score, which evaluates features based on their ability to preserve local structures [29]. Hu et al. leverage a Laplacian-based graph regularizer within a feature-level self-representation framework to capture local manifold structure and conduct unsupervised feature selection via $\ell_{2,1}$-norm sparsity [30]. Cai et al. leverage the Laplacian matrix to capture the multi-cluster structure of data and effectively select features preserving the data’s intrinsic manifold structure through sparse regression [31]. However, graph-based Laplacian regularization is frequently employed to preserve local geometric structures by enforcing smoothness over similarity graphs, which leads to the fact that Laplacian regularization mainly captures the pairwise linear relationships between samples, and cannot model high-order geometric attributes such as manifold curvature or topological connectivity [32,33].

Hessian regularization offers superior extrapolation capabilities compared to Laplacian regularization because of its broader null space. This regularization prefers functions that undergo linear transformations based on geodesic distance, thus effectively retaining the data’s topological structure [34]. The Hessian locally linear embedding technique employs second-order derivative constraints to maintain manifold curvature and effectively captures the intrinsic curvature as well as the topological connectivity of data manifolds [35]. Sheikhpour et al. employ a generalized uncorrelated constraint with mixed $\ell_{2,p}$-norm regularization, leveraging Hessian regularization to preserve the data’s topological structure [36]. Although Hessian-based graph learning is often regarded as particularly suitable for semi-supervised settings, where limited label information can guide structure preservation, its ability to capture high-order local geometry also makes it useful for unsupervised representation learning. Moslemi et al. effectively preserve the geometrical structure of data and select the most discriminative features by utilizing Hessian regularized latent representation learning with an $\ell_{2,0}$-norm constraint [17].

In addition, low-rank approximation seeks to capture the global correlation structure among features, making it an important method in feature selection. Wang et al. tackle the feature selection problem by employing matrix factorization coupled with low-rank constraints, thereby capitalizing on the benefits of both the low-rank characteristics and the structure learning aspects [20]. Zheng et al. learn a data dictionary with low-rank constraints and perform feature reconstruction to effectively reduce redundant features and noise while preserving the sample distribution in multiple subspaces [37]. Chen et al. minimize the weighted distance between simplified matrix representations to address issues in existing methods that neglect the local structure of sample data [38].

Besides classical model-driven unsupervised feature selection, recent studies have explored deep unsupervised feature selection by coupling feature selection with latent representation learning. Han et al. propose an autoencoder-inspired unsupervised feature selection method that combines autoencoder regression with group lasso regularization, enabling the model to identify informative features by exploiting nonlinear relationships among features [23]. Moslemi and Jamshidi develop an autoencoder-based unsupervised feature selection framework with sparse manifold learning, in which both the encoding and decoding processes are regularized and the geometric structure of the input data is preserved through Laplacian graph constraints [39]. Song et al. propose a dual-graph autoencoder for unsupervised feature selection, which incorporates graph regularization in both the data space and feature space while imposing sparsity-promoting norm constraints on the encoder and decoder to improve robustness and structural preservation [40]. These methods generally integrate autoencoder-based reconstruction, manifold preservation, and graph regularization into neural architectures, thereby enhancing nonlinear representation learning and improving feature discrimination.

## 3. The proposed method

### 3.1 Notations

To start, we review some essential symbols that will be consistently applied in this paper. For a column vector $𝐯=(v_{1},...,v_{n}{)}^{T}\in {\mathbb{R}}^{n}$, its Euclidean norm is defined as $\|𝐯{\|}_{2}=\sqrt{\sum_{i=1}^{n}v_{i}^{2}}$. For an arbitrary matrix $𝐌=({𝐌}_{ij}{)}_{n\times m}\in {\mathbb{R}}^{n\times m}$, the $\ell_{2,1}$-norm is given by $\|𝐌{\|}_{2,1}=\sum_{i=1}^{n}\sqrt{\sum_{j=1}^{m}{𝐌}_{ij}^{2}}$. The rank operator rank(·) applied to 𝐌 returns its matrix rank. When 𝐌 represents a square matrix in ${\mathbb{R}}^{n\times n}$, its trace is computed as $\text{Tr}(𝐌)=\sum_{i=1}^{n}{𝐌}_{ii}$.

### 3.2 Problem formulation

We denote the data matrix as $𝐗=({𝐱}_{1};...;{𝐱}_{n})=({𝐟}_{1},...,{𝐟}_{d})\in {\mathbb{R}}^{n\times d}$ where ${𝐟}_{j}\in {\mathbb{R}}^{n}$ is a feature vector and ${𝐱}_{i}\in {\mathbb{R}}^{d}$ is a sample. Employing the regularized self-representation of the data matrix [24], the unsupervised feature selection problem can be mathematically expressed as


$$\begin{array}{cc} & \underset{𝐖}{\mathrm{argmin}}\|𝐗-𝐗𝐖{\|}_{2,1}+\gamma \|𝐖{\|}_{2,1} \end{array}$$ (1)

where $𝐖=({𝐰}_{1};\cdots ;{𝐰}_{d})$, ${𝐰}_{i}$ is a *d*-dimensional column vector, and γ is a parameter that balances the accuracy of the model fit with the regularization strength. Here, $𝐖\in {\mathbb{R}}^{d\times d}$ denotes the coefficient matrix used to identify the selected features, the term $\|𝐖{\|}_{2,1}$ acts as a regularization factor aimed at promoting sparsity and addressing outliers, while $\|{𝐰}_{i}{\|}_{2}$ represents the significance of the *i*-th feature. A higher value of $\|{𝐰}_{i}{\|}_{2}$ indicates a higher importance of the corresponding feature.

### 3.3 Low-rank approximation

To explicitly reduce feature redundancy and avoid degenerate self-representation solutions, we impose a low-rank constraint on the coefficient matrix 𝐖. It can not only identify latent correlations among features but also help avoid degenerate or trivial solutions in the self-representation model. The fundamental objective of unsupervised feature selection lies in identifying a compact subset of features that optimally preserves the essential characteristics of the initial high-dimensional space. The low-rank approximation is imposed on the coefficient matrix 𝐖 to promote a compact and informative low-dimensional structure for feature selection [20]. Then we can obtain the following model:


$$\begin{array}{cc} & \underset{𝐖}{\mathrm{argmin}}\|𝐗-𝐗𝐖{\|}_{2,1}+\gamma \|𝐖{\|}_{2,1}\\ & \text{s.t. rank}(𝐖)=l \end{array}$$ (2)

where *l* denotes the number of selected features, and the condition rank(𝐖)=l imposes a low-rank constraint ensuring that 𝐖 serves as a pseudo-indicator matrix for feature selection. By explicitly incorporating the rank constraint into the optimization objective, the learned coefficient matrix is encouraged to exhibit a compact structure for feature selection.

### 3.4 Hessian regularization

Structure learning plays a pivotal role in addressing unsupervised learning challenges. Nonetheless, the aforementioned optimization problem overlooks the inherent structural characteristics of the input data. Motivated by the principles of manifold learning, we integrate a manifold regularization term to preserve local similarity within the data [17,36]. It solves the problem of Laplacian regularization being unable to effectively preserve complex topological structures and nonlinear geometric information of data due to null-space constraints.

Following standard Hessian regularization, we introduce a local geometric smoothness assumption in constructing the regularizer. This assumption is introduced as a local approximation for neighborhood structure learning, rather than a global smooth-manifold requirement on the data.

Let $M\subset R^{d}$ denote a manifold. The Hessian energy for the mapping f:M→ℝ is defined as:


$$\begin{array}{c} E_{𝐇}(f)=\int_{M}{\left\|\nabla_{a}\nabla_{b}f\right\|}_{T_{𝐱}^{*}M⊗T_{𝐱}^{*}M}^{2}dV(𝐱) \end{array}$$ (3)

where $T_{x}^{*}M$ represents the local cotangent space at point 𝐱∈M, *f* is the mapping function, and $\nabla_{a}\nabla_{b}f$ denotes the second covariant derivative of *f*. The term dV(𝐱) signifies the natural volume element on the manifold *M*. Since the Hessian energy depends on the manifold geometry, it can be locally evaluated using normal coordinates on M. The normal coordinates for a designated point 𝐱 are defined as the coordinates on *M* such that the manifold is locally approximated as Euclidean around *x*. Consequently, normal coordinates ${𝐱}^{r}$ and ${𝐱}^{s}$ centered at 𝐱 are derived through the following methodology:


$$\begin{array}{c} {\left\|\nabla_{a}\nabla_{b}f\right\|}_{T_{x}^{*}M⊗T_{x}^{*}M}^{2}=\sum_{r,s=1}^{m}{\left(\frac{\partial^{2}f}{\partial {𝐱}^{r}\partial {𝐱}^{s}}\right)}^{2} \end{array}$$ (4)

Eq. (4) elucidates that at the point 𝐱, the norm of the second covariant derivative corresponds to the Frobenius norm of the Hessian matrix of *f* within the normal coordinates.

We utilize the *k* nearest neighbors $\left({ℵ}_{k}\left({𝐱}_{i}\right)\right)$ to establish the local normal coordinate system. Then, the local tangent space $T_{𝐱}^{*}M$ is approximated using the PCA technique, where the top *m* eigenvectors serve as the estimated orthogonal basis for $T_{𝐱}^{*}M$. Following the classical Hessian regularization construction, this step provides a local linear approximation of the underlying manifold. Therefore, its quality depends on the neighborhood quality: when the neighborhood samples lie close to a low-dimensional manifold, the approximation is reliable; however, heavy noise or severe outlier contamination may deteriorate the estimated tangent space and consequently affect the resulting Hessian matrix. Consequently, the normal coordinates ${𝐱}^{r}$ and ${𝐱}^{s}$ of a neighboring point ${𝐱}_{q}\in {ℵ}_{k}\left({𝐱}_{i}\right)$, for 1≤q≤k, can be approximated through the local tangent space $T_{𝐱}^{*}M$. The Hessian of *f* at ${𝐱}_{i}$ is approximated as described below:


$$\begin{array}{c} {\frac{\partial^{2}f}{\partial {𝐱}^{r}\partial {𝐱}^{s}}|}_{{𝐱}_{i}}\approx \sum_{q=1}^{k}Z_{rsq}^{\left(i\right)}f\left({𝐱}_{q}\right) \end{array}$$ (5)

where $Z_{rsq}^{\left(i\right)}$ represents the local Hessian operator for the sample ${𝐱}_{i}$ in the normal coordinates ${𝐱}^{r}$ and ${𝐱}^{s}$ of a point ${𝐱}_{q}\in {ℵ}_{k}({𝐱}_{i})$. The operator $Z_{rsq}^{\left(i\right)}$ can be determined by fitting a second-order polynomial p(𝐱) in these normal coordinates to the values $f({𝐱}_{q})$ for q=1,...,k. Moreover, following the Hessian-energy estimator in [41], we fix the zeroth-order term at $f({𝐱}_{i})$ in the local second-order polynomial fitting, which improves the stability of the subsequent Hessian estimation and helps avoid degenerate polynomial fits, as detailed below:


$$\begin{array}{c} p^{i}(𝐱)=f\left({𝐱}_{i}\right)+\sum_{r=1}^{m}{𝐇}_{r}{𝐱}^{r}+\sum_{r=1}^{m}\sum_{s=r}^{m}{𝐍}_{rs}{𝐱}^{r}{𝐱}^{s} \end{array}$$ (6)

where $f({𝐱}_{i})$ denotes the zeroth-order term, ${𝐇}_{r}$ denotes the first-order coefficient, and ${𝐍}_{rs}$ denotes the second-order coefficient. Eq. (6) can be interpreted as the second-order Taylor expansion of *f* centered at ${𝐱}_{i}$, as the neighborhood size diminishes towards zero. Subsequently, ${𝐇}_{r}$ and ${𝐍}_{rs}$ are:


$$\begin{array}{c} {𝐇}_{r}=\frac{\partial f}{\partial {𝐱}^{r}}{|}_{{𝐱}_{i}},\;{𝐍}_{rs}=\frac{1}{2}\frac{\partial^{2}f}{\partial {𝐱}^{r}\partial {𝐱}^{s}}{|}_{{𝐱}_{i}} \end{array}$$ (7)

with ${𝐍}_{rs}={𝐍}_{sr}$. In order to estimate the value of the function at point ${𝐱}_{i}$ and its adjacent points by using the basis function in the local coordinate system, and then calculate the local Hessian matrix, we utilize the method of ordinary least squares to fit the polynomial as depicted in Eq. (8).


$$\begin{array}{c} \underset{𝐯\in R^{p}}{\mathrm{argmin}}\sum_{q=1}^{k}{\left(\left(f\left({𝐱}_{q}\right)-f\left({𝐱}_{i}\right)\right)-(\Phi 𝐯{)}_{q}\right)}^{2} \end{array}$$ (8)

where $\Phi \in {\mathbb{R}}^{k\times p}$ represents the design matrix with $p=m+\frac{m(m+1)}{2}$. The corresponding basis functions φ consist of the monomials of the normal coordinates (centered at ${𝐱}_{i}$) of ${𝐱}_{q}\in {ℵ}_{k}({𝐱}_{i})$ up to the second order. The solution to Eq. (8) is given by $𝐯=\Phi^{†}𝐰$, where $𝐰=(w_{1},w_{2},...,w_{k}{)}^{T}\in {\mathbb{R}}^{k}$, $w_{q}=f({𝐱}_{q})$, and † denotes the Moore-Penrose inverse.

To avoid confusion, we assume $f({𝐱}_{\pi })=f_{\pi }$ and $f({𝐱}_{\mu })=f_{\mu }$. The Frobenius norm of the Hessian of *f* at ${𝐱}_{i}$ is approximated by the following expression:


$$\begin{array}{c} \|\nabla_{a}\nabla_{b}f{\|}^{2}\approx \sum_{r,s=1}^{n}{\left(\sum_{\pi =1}^{k}Z_{rs\pi }^{\left(i\right)}f_{\pi }\right)}^{2}=\sum_{\pi ,\mu =1}^{k}f_{\pi }f_{\mu }{𝐇}_{\pi \mu }^{\left(i\right)} \end{array}$$ (9)

where ${𝐇}_{\pi \mu }^{\left(i\right)}=\sum_{r,s=1}^{n}Z_{rs\pi }^{\left(i\right)}Z_{rs\mu }^{\left(i\right)}$. Ultimately, the Hessian energy function is approximated by the following formula:


$$\begin{array}{c} S_{𝐇}(f)=\sum_{i=1}^{n}\sum_{{𝐱}_{\pi }\in {ℵ}_{k}({𝐱}_{i})}\sum_{{𝐱}_{\mu }\in {ℵ}_{k}({𝐱}_{i})}f_{\pi }f_{\mu }{𝐇}_{\pi \mu }^{\left(i\right)}={𝐘}^{T}𝐇𝐘 \end{array}$$ (10)

where $𝐇=\sum_{i=1}^{n}{𝐇}^{\left(i\right)}$ and 𝐘 represents the data in the local tangent space.

Then, 𝐘 can be expressed through 𝐖 and 𝐗, that is, 𝐘=XW. Here, W is the coefficient matrix that maps the original feature space 𝐗 to the learned representation space. Substituting 𝐘=XW into the Hessian energy function, we obtain $S_{𝐇}=\mathrm{Tr}\left({𝐖}^{T}{𝐗}^{T}HXW\right)$. It is worth noting that the effectiveness of this regularizer depends on the quality of local neighborhood approximation. In cases where the local geometry is highly irregular or non-smooth, the Hessian term may offer weaker structural characterization, although the overall framework remains applicable.

Incorporating the Hessian regularization into our framework Eq. (2), the final objective function is presented as follows:


$$\begin{array}{cc} & \underset{𝐖}{\mathrm{argmin}}\|X-XW{\|}_{2,1}+\alpha \text{Tr}({𝐖}^{T}{𝐗}^{T}HXW)+\gamma \|𝐖{\|}_{2,1}\\ & \text{s.t. rank}(𝐖)=l \end{array}$$ (11)

where α is a coefficient that balances local geometric-structure preservation and reconstruction error.

## 4. Optimization and algorithm

### 4.1 Optimization

Utilizing the property that $\text{rank}(𝐖)=\text{rank}({WW}^{T})$, and given that ${WW}^{T}$ is positive semi-definite, the magnitude of $\text{rank}({WW}^{T})$ corresponds to the number of non-negative eigenvalues of ${WW}^{T}$. Therefore, based on $\text{rank}(𝐖)=\text{rank}({WW}^{T})=l$, it follows that ${WW}^{T}$ has d−l eigenvalues equal to zero. Let $\sigma_{i}(𝐋)$ denote the *i*-th singular value of matrix 𝐋. We subsequently use the expression $\beta \sum_{i=1}^{d-l}\sigma_{i}(𝐖{𝐖}^{T})$ to approximate the rank of $𝐖{𝐖}^{T}$. By penalizing the sum of the d−l smallest eigenvalues of ${WW}^{T}$, the low-rank constraint in Eq. (11) is relaxed into a penalized approximation. In accordance with Ky Fan’s Theorem [42], we introduce a variable $𝐅\in {\mathbb{R}}^{d\times (d-l)}$ to approximate the sum of the d−l smallest singular values, thus ultimately leading to the following relationship:


$$\begin{array}{c} \sum_{i=1}^{d-l}\sigma_{i}({WW}^{T})=\min_{{𝐅}^{T}F=I}\text{Tr}({𝐅}^{T}{WW}^{T}𝐅) \end{array}$$ (12)

Employing the method outlined above to convert the low-rank constraint into an objective function for optimization, Eq. (11) can be reformulated as follows:


$$\begin{array}{cc} & \underset{𝐖}{\mathrm{argmin}}\|X-XW{\|}_{2,1}+\alpha \text{Tr}({𝐖}^{T}{𝐗}^{T}HXW)+\beta \mathrm{Tr}\left({𝐅}^{T}{WW}^{T}𝐅\right)+\gamma \|𝐖{\|}_{2,1}\\ & \text{ s.t. }{𝐅}^{T}𝐅=𝐈 \end{array}$$ (13)

#### 4.1.1 Update 𝐅 with fixed 𝐖.

The optimization problem mentioned above, concerning 𝐅, is equivalently reformulated in the subsequent expression:


$$\begin{array}{c} \underset{𝐅}{\mathrm{argmin}}\beta Tr\left({𝐅}^{T}{WW}^{T}𝐅\right)\text{ s.t. }{𝐅}^{T}𝐅=𝐈 \end{array}$$ (14)

To address this, we apply the Lagrange multiplier method to this constrained optimization problem:


$$\begin{array}{c} L(F,A)=\text{Tr}({𝐅}^{T}{WW}^{T}𝐅)+\lambda \text{Tr}[𝐀({𝐅}^{T}𝐅-𝐈)] \end{array}$$ (15)

where $A\in {\mathbb{R}}^{\left(d-l)\times (d-l\right)}$ is the Lagrange multiplier matrix. Next, we compute the derivatives with respect to 𝐅 and 𝐀:


$$\begin{array}{c} \left\{ \begin{array}{c} \frac{\partial L}{\partial 𝐅}=2{WW}^{T}𝐅+2\lambda 𝐀\\ \frac{\partial L}{\partial 𝐀}=\lambda ({𝐅}^{T}𝐅-𝐈) \end{array}\right. \end{array}$$ (16)

Setting $\frac{\partial L}{\partial 𝐅}=0$ and $\frac{\partial L}{\partial 𝐀}=0$ yields the optimal solution $𝐅=-\lambda {\left(𝐖{𝐖}^{T}\right)}^{-1}𝐀$ and the orthogonality of ${𝐅}^{T}𝐅=𝐈$.

#### 4.1.2 Update 𝐖 with fixed 𝐅.

Owing to the non-smooth nature of the $\ell_{2,1}$-norm, the iterative reweighted least-squares (IRLS) algorithm was introduced to tackle this issue [43,44]. We define the diagonal weighting matrices $𝐃\in {\mathbb{R}}^{d\times d}$ and $𝐆\in {\mathbb{R}}^{n\times n}$, where $𝐃=\text{diag}\left(\frac{1}{2\|{𝐰}_{1}{\|}_{2}},\frac{1}{2\|{𝐰}_{2}{\|}_{2}},...,\frac{1}{2\|{𝐰}_{d}{\|}_{2}}\right)$ and $𝐆=\mathrm{diag}\left(\frac{1}{2{\left\|(𝐗-𝐗𝐖{)}_{1}\right\|}_{2}},\frac{1}{2{\left\|(𝐗-XW{)}_{2}\right\|}_{2}},...,\frac{1}{2{\left\|(𝐗-XW{)}_{n}\right\|}_{2}}\right)$.

Furthermore, according to the operational properties of matrix trace $\text{Tr}(M_{1}M_{2})=\text{Tr}(M_{2}M_{1})$, we have


$$\begin{array}{c} \text{Tr}({𝐅}^{T}{WW}^{T}𝐅)=\text{Tr}({𝐖}^{T}𝐅{𝐅}^{T}𝐖) \end{array}$$ (17)

Subsequently, ${𝐖}^{t+1}$ is updated by addressing the following weighted least squares issue:


$$\begin{array}{cc} {𝐖}^{t+1}=\underset{𝐖}{\mathrm{argmin}} & \mathrm{Tr}\left((𝐗-XW{)}^{T}{𝐆}^{t}\left(X-XW\right)\right)+\alpha \mathrm{Tr}\left({𝐖}^{T}{𝐗}^{T}HXW\right)+\beta \mathrm{Tr}\left({𝐖}^{T}{FF}^{T}𝐖\right)\\ & +\gamma \mathrm{Tr}\left({𝐖}^{T}{𝐃}^{t}𝐖\right)\\ =\underset{𝐖}{\mathrm{argmin}} & \mathrm{Tr}\left((𝐗-XW{)}^{T}{𝐆}^{t}\left(X-XW\right)\right)+\mathrm{Tr}\left({𝐖}^{T}\left(\alpha {𝐗}^{T}HX+\beta 𝐅{𝐅}^{T}+\gamma {𝐃}^{t}\right)𝐖\right) \end{array}$$ (18)

The closed-form solution for ${𝐖}^{t+1}$ is given by


$$\begin{array}{cc} {𝐖}^{t+1} & ={\left({𝐗}^{T}{𝐆}^{t}𝐗+\alpha {𝐗}^{T}HX+\beta {FF}^{T}+\gamma {𝐃}^{t}\right)}^{-1}{𝐗}^{T}{𝐆}^{t}𝐗\\ & =\left[({𝐃}^{t}{)}^{-1}{𝐗}^{T}{𝐆}^{t}𝐗+\alpha ({𝐃}^{t}{)}^{-1}{𝐗}^{T}HX+\beta ({𝐃}^{t}{)}^{-1}{FF}^{T}+\gamma 𝐈\right]({𝐃}^{t}{)}^{-1}{𝐗}^{T}{𝐆}^{t}𝐗 \end{array}$$ (19)

where $𝐈\in {\mathbb{R}}^{d\times d}$ denotes the identity matrix. A sufficiently small value ϵ is incorporated by defining ${𝐆}_{ii}^{t}=1/\max(2\|{𝐱}_{i}-{𝐱}_{i}{𝐖}^{t}{\|}_{2},\epsilon )$ and ${𝐃}_{jj}^{t}=1/\max(2\|{𝐰}_{j}^{t}{\|}_{2},\epsilon )$ to avoid division by zero.

### 4.2 Algorithm and computational complexity analysis

The preceding discussion has addressed each variable under optimization individually. Building on these analyses, the procedure for our method is encapsulated in Algorithm 1.

**Algorithm 1. Hessian-based Unsupervised Feature Selection using Low-Rank Approximation (HLR-UFS)**

**Input:** Data matrix $𝐗\in {\mathbb{R}}^{n\times d}$, target feature number *l*, parameters α,β,γ.

**Output:** Selected feature subset *I*.

1 Initialize 𝐖;

2 Construct Hessian regularization matrix 𝐇 via Eq. (10).

**repeat**

3 Update 𝐅 as eigenvectors of ${WW}^{T}$ corresponding to the smallest d−l eigenvalues.

4 Compute diagonal matrices 𝐆 (${𝐆}_{ii}=\frac{1}{2\|(𝐗-XW{)}_{i}{\|}_{2}}$) and 𝐃 (${𝐃}_{ii}=\frac{1}{2\|{𝐰}_{i}{\|}_{2}}$).

5 Update $𝐖\leftarrow {\left({𝐗}^{T}GX+\alpha {𝐗}^{T}HX+\beta {FF}^{T}+\gamma 𝐃\right)}^{-1}{𝐗}^{T}GX$.

**until** Convergence

6 Sort $\|{𝐰}_{i}{\|}_{2}$ (i∈[d]) in a descending sequence and select top-*l* indices as *I*.

The proposed HLR-UFS algorithm involves two key subproblems: updating the coefficient matrix 𝐖 and the low-rank projection matrix 𝐅. The time complexity of each iteration is dominated by matrix operations.

- **Updating** 𝐅: To solve the orthogonal constraint problem via eigenvalue decomposition of $𝐖{𝐖}^{T}$, the time complexity is *O*(*d*^3^) per iteration.
- **Updating** 𝐖: The predominant computational cost rests in calculating the inverse of a d×d matrix in Eq. (19). Using the Woodbury identity, the complexity is reduced from *O*(*d*^3^) to $O(\min(n,d{)}^{3})$. Overall, the complexity for updating 𝐖 is $O(\min(n,d{)}^{3}+nd^{2})$.

Combining these steps, each iteration of the HLR-UFS algorithm has the time complexity of $O(d^{3}+\min(n,d{)}^{3}+nd^{2})$. The overall complexity depends on the number of iterations *T* needed for convergence, thereby yielding a total time complexity of $O(T(d^{3}+\min(n,d{)}^{3}+nd^{2}))$.

## 5. Convergence analysis

In this section, we theoretically analyze the convergence of the proposed HLR-UFS algorithm. Let L(𝐖,𝐅) denote the original objective function:


$$\begin{array}{cc} L(𝐖,𝐅)= & \;\|𝐗-𝐗𝐖{\|}_{2,1}+\alpha \mathrm{Tr}({𝐖}^{T}{𝐗}^{T}𝐇𝐗𝐖)\\ & +\beta \mathrm{Tr}({𝐖}^{T}𝐅{𝐅}^{T}𝐖)+\gamma \|𝐖{\|}_{2,1}. \end{array}$$ (20)

To handle the non-smoothness of *L*, we construct the following surrogate function *J* at the *t*-th iteration:


$$\begin{array}{cc} & J(𝐖,{𝐅}^{t}|{𝐖}^{t})\\ & =\mathrm{Tr}\left((𝐗-𝐗𝐖{)}^{T}{𝐆}^{t}(𝐗-𝐗𝐖)\right)+\alpha \mathrm{Tr}({𝐖}^{T}{𝐗}^{T}𝐇𝐗𝐖)\\ & \;+\beta \mathrm{Tr}({𝐖}^{T}{𝐅}^{t}({𝐅}^{t}{)}^{T}𝐖)+\gamma \mathrm{Tr}({𝐖}^{T}{𝐃}^{t}𝐖), \end{array}$$ (21)

where ${𝐆}^{t}$ and ${𝐃}^{t}$ are diagonal matrices with their diagonal entries defined as:


$$\begin{array}{c} {𝐆}_{ii}^{t}=\frac{1}{2\|(𝐗-𝐗{𝐖}^{t}{)}_{i}{\|}_{2}},\;{𝐃}_{jj}^{t}=\frac{1}{2\|{𝐰}_{j}^{t}{\|}_{2}}. \end{array}$$

**Theorem 1.** $J(𝐖,{𝐅}^{t}|{𝐖}^{t})$ *is a surrogate function for* $L(𝐖,{𝐅}^{t})$*. More specifically, for any* 𝐖*, we have* $L(𝐖,{𝐅}^{t})\leq J(𝐖,{𝐅}^{t}|{𝐖}^{t})$*, and the equality holds if and only if* $𝐖={𝐖}^{t}$.

*Proof of Theorem 1.* For any vector 𝐳 and any nonzero vector ${𝐳}^{t}$, the following inequality holds:


$$\begin{array}{c} \|𝐳{\|}_{2}\leq \frac{\|𝐳{\|}_{2}^{2}}{2\|{𝐳}^{t}{\|}_{2}}+\frac{\|{𝐳}^{t}{\|}_{2}}{2}, \end{array}$$ (22)

where equality holds when $𝐳={𝐳}^{t}$.

We apply the inequality in Eq. (22) to each row of the residual matrix 𝐗−𝐗𝐖. Let ${𝐱}_{i}-{𝐱}_{i}𝐖$ and ${𝐱}_{i}-{𝐱}_{i}{𝐖}^{t}$ denote the *i*-th row of 𝐗−𝐗𝐖 and $𝐗-𝐗{𝐖}^{t}$, respectively. Then for each row i=1,...,n, we have:


$$\begin{array}{c} \|{𝐱}_{i}-{𝐱}_{i}𝐖{\|}_{2}\leq \frac{\|{𝐱}_{i}-{𝐱}_{i}𝐖{\|}_{2}^{2}}{2\|{𝐱}_{i}-{𝐱}_{i}{𝐖}^{t}{\|}_{2}}+\frac{\|{𝐱}_{i}-{𝐱}_{i}{𝐖}^{t}{\|}_{2}}{2}. \end{array}$$ (23)

Similarly, applying the inequality to each row of the coefficient matrix W gives:


$$\begin{array}{c} \|{𝐰}_{j}{\|}_{2}\leq \frac{\|{𝐰}_{j}{\|}_{2}^{2}}{2\|{𝐰}_{j}^{t}{\|}_{2}}+\frac{\|{𝐰}_{j}^{t}{\|}_{2}}{2}. \end{array}$$ (24)

Let $F(𝐖)=L(𝐖,{𝐅}^{t})-J(𝐖,{𝐅}^{t}|{𝐖}^{t})$. In the following, we will prove that for any 𝐖, there is $F({𝐖}^{t})-F(𝐖)\geq 0$. First, $F({𝐖}^{t})$ can be evaluated as:


$$\begin{array}{cc} F({𝐖}^{t})=\; & \sum_{i=1}^{n}\|{𝐱}_{i}-{𝐱}_{i}{𝐖}^{t}{\|}_{2}+\gamma \sum_{j=1}^{d}\|{𝐰}_{j}^{t}{\|}_{2}\\ & -\left(\sum_{i=1}^{n}\frac{\|{𝐱}_{i}-{𝐱}_{i}{𝐖}^{t}{\|}_{2}^{2}}{2\|{𝐱}_{i}-{𝐱}_{i}{𝐖}^{t}{\|}_{2}}+\gamma \sum_{j=1}^{d}\frac{\|{𝐰}_{j}^{t}{\|}_{2}^{2}}{2\|{𝐰}_{j}^{t}{\|}_{2}}\right)\\ =\; & \frac{1}{2}\sum_{i=1}^{n}\|{𝐱}_{i}-{𝐱}_{i}{𝐖}^{t}{\|}_{2}+\frac{\gamma }{2}\sum_{j=1}^{d}\|{𝐰}_{j}^{t}{\|}_{2}. \end{array}$$ (25)

Then, $F({𝐖}^{t})-F(𝐖)$ can be rewritten as:


$$\begin{array}{cc} & F({𝐖}^{t})-F(𝐖)\\ = & \frac{1}{2}\left(\sum_{i=1}^{n}\|{𝐱}_{i}-{𝐱}_{i}{𝐖}^{t}{\|}_{2}+\gamma \sum_{j=1}^{d}\|{𝐰}_{j}^{t}{\|}_{2}\right)\\ & -(\sum_{i=1}^{n}\|{𝐱}_{i}-{𝐱}_{i}𝐖{\|}_{2}+\gamma \sum_{j=1}^{d}\|{𝐰}_{j}{\|}_{2}\\ & -\sum_{i=1}^{n}\frac{\|{𝐱}_{i}-{𝐱}_{i}𝐖{\|}_{2}^{2}}{2\|{𝐱}_{i}-{𝐱}_{i}{𝐖}^{t}{\|}_{2}}-\gamma \sum_{j=1}^{d}\frac{\|{𝐰}_{j}{\|}_{2}^{2}}{2\|{𝐰}_{j}^{t}{\|}_{2}})\\ = & \sum_{i=1}^{n}\frac{{\left(\|{𝐱}_{i}-{𝐱}_{i}{𝐖}^{t}{\|}_{2}-\|{𝐱}_{i}-{𝐱}_{i}𝐖{\|}_{2}\right)}^{2}}{2\|{𝐱}_{i}-{𝐱}_{i}{𝐖}^{t}{\|}_{2}}\\ & +\gamma \sum_{j=1}^{d}\frac{{\left(\|{𝐰}_{j}^{t}{\|}_{2}-\|{𝐰}_{j}{\|}_{2}\right)}^{2}}{2\|{𝐰}_{j}^{t}{\|}_{2}}. \end{array}$$ (26)

Because $2\|{𝐱}_{i}-{𝐱}_{i}{𝐖}^{t}{\|}_{2}\geq 0$ and $2\|{𝐰}_{j}^{t}{\|}_{2}\geq 0$ (in practice, an infinitesimal constant is added to avoid division by zero), and the numerators are perfect squares, we have $F({𝐖}^{t})-F(𝐖)\geq 0$. Consequently, *J* is a valid surrogate function satisfying $L(𝐖,{𝐅}^{t})\leq J(𝐖,{𝐅}^{t}|{𝐖}^{t})$.

**Theorem 2.** *The proposed HLR-UFS algorithm yields a monotonically non-increasing original objective function* L(𝐖,𝐅) *in each iteration, i.e.,:*


$$\begin{array}{c} L({𝐖}^{t+1},{𝐅}^{t+1})\leq L({𝐖}^{t},{𝐅}^{t}). \end{array}$$ (27)

*Proof of Theorem 2.* The alternating minimization process can be decomposed into two steps. We will prove that neither updating 𝐖 nor updating 𝐅 will increase the objective function.

**Step 1: Updating** 𝐖**.** Given ${𝐅}^{t}$, the *t*-th iteration aims to solve ${𝐖}^{t+1}=\underset{𝐖}{\mathrm{argmin}}J(𝐖,{𝐅}^{t}|{𝐖}^{t})$. Because ${𝐖}^{t+1}$ is the minimizer of *J*, we have $J({𝐖}^{t+1},{𝐅}^{t}|{𝐖}^{t})\leq J({𝐖}^{t},{𝐅}^{t}|{𝐖}^{t})$. Combining this with Theorem 1, we ob*t*ain:
  $$\begin{array}{c} L({𝐖}^{t+1},{𝐅}^{t})\leq J({𝐖}^{t+1},{𝐅}^{t}|{𝐖}^{t})\leq J({𝐖}^{t},{𝐅}^{t}|{𝐖}^{t})=L({𝐖}^{t},{𝐅}^{t}). \end{array}$$ (28)

Thus, updating 𝐖 does not increase the objective function.

**Step 2: Updating** 𝐅**.** With ${𝐖}^{t+1}$ fixed, ${𝐅}^{t+1}$ is obtained by solving the orthogonality-constrained problem:
  $$\begin{array}{c} {𝐅}^{t+1}=\underset{{𝐅}^{T}𝐅=𝐈}{\text{arg}\min}Tr\left({𝐅}^{T}{𝐖}^{t+1}({𝐖}^{t+1}{)}^{T}𝐅\right). \end{array}$$

This is a standard eigenvalue problem, and its closed-form solution is given by the eigenvectors corresponding to the d−l smallest eigenvalues of ${𝐖}^{t+1}({𝐖}^{t+1}{)}^{T}$. Due to the optimality of ${𝐅}^{t+1}$, we have:


$$\begin{array}{c} Tr\left(({𝐅}^{t+1}{)}^{T}{𝐖}^{t+1}({𝐖}^{t+1}{)}^{T}{𝐅}^{t+1}\right)\leq Tr\left(({𝐅}^{t}{)}^{T}{𝐖}^{t+1}({𝐖}^{t+1}{)}^{T}{𝐅}^{t}\right). \end{array}$$ (29)

Since the other terms in *L* are independent of 𝐅, it yields:


$$\begin{array}{c} L({𝐖}^{t+1},{𝐅}^{t+1})\leq L({𝐖}^{t+1},{𝐅}^{t}). \end{array}$$

**Step 3: Summary.** Combining the results from Step 1 and Step 2, we arrive at:
  $$\begin{array}{c} L({𝐖}^{t+1},{𝐅}^{t+1})\leq L({𝐖}^{t+1},{𝐅}^{t})\leq L({𝐖}^{t},{𝐅}^{t}), \end{array}$$ (30)

where the first inequality stems from the fact that $J(𝐖,{𝐅}^{t}|{𝐖}^{t})$ is a surrogate function, and the second inequality results from the optimality of ${𝐖}^{t+1}=\underset{𝐖}{\mathrm{argmin}}J(𝐖,{𝐅}^{t}|{𝐖}^{t})$.□

## 6. Experiments

Since HLR-UFS is an optimization-based unsupervised feature selection method, the main experimental comparisons are conducted against representative non-deep baselines under a unified evaluation framework in which clustering performance is assessed on the selected features.

### 6.1 Datasets

Experiments have been carried out across nine distinct benchmark datasets: the COIL20 object image dataset, the Arcene mass spectrometry dataset, the USPS digit image dataset, and four facial image datasets (warpAR10P, JAFFE, orlraws10P, and Yale_32x32), as well as two biomedical datasets (TOX-171 and Prostate-GE). The characteristics of these datasets are summarized in Table 1. Given their diverse data sources, these datasets provide an effective platform for thoroughly evaluating the performance of various methods.

**Table 1:** Data description.

DID	datasets	# instances	# features	# classes	data types
1	warpAR10P	130	2400	10	Face images
2	JAFFE	213	676	10	Face images
3	Yale_32x32	165	1024	15	Face images
4	orlraws10P	100	10304	10	Face images
5	COIL20	1440	1024	20	Object images
6	USPS	9298	256	10	Digit image
7	TOX-171	171	5748	4	Biology
8	Prostate-GE	102	5966	2	Biology
9	arcene	200	10000	2	Mass spectrometry

### 6.2 Comparison algorithms

We compare the proposed method with several state-of-the-art unsupervised feature selection methods to demonstrate its effectiveness and efficiency.

- **Baseline**: All original features are used without feature selection.
- **LS** [29]: It relies on Laplacian eigenmaps and the projection that maintains localities, and it assesses the features by their ability to preserve locality.
- **RUFS** [45]: It integrates robust non-negative matrix factorization with local learning regularization and employs joint $\ell_{2,1}$-norm minimization to enhance feature selection performance.
- **LRSL** [20]: It formulates feature selection as a matrix decomposition problem, where low-rank constraints and graph-based Laplacian regularization are jointly optimized.
- **MRSR** [46]: It preserves local data structures via $\ell_{2,1}$-norm reconstruction and sparsity constraints, optimized by iterative reweighted least squares.
- **SLNMF** [47]: It uses convex nonnegative matrix factorization to reduce dimensionality and enhance sparsity, while soft-label learning guides the feature selection process by capturing the ambiguous associations between data points and the centroids of clusters.
- **RAFG** [48]: An unsupervised feature selection method using adaptive $\ell_{1}$-norm graph learning. It employs $\ell_{2,1}$-norm regression and $\ell_{2,p}$-norm sparsity for noise-robust feature ranking and structure preservation.
- **LRPFS** [26]: It introduces a new latent relationship penalty term to preserve the data structure and uniqueness of samples, and employs an $\ell_{2,1}$-norm sparsity constraint to enhance computational efficiency.

### 6.3 Parameter setting

In order to evaluate the efficacy of different feature selection methods, it is necessary to set certain parameters beforehand. First, for our proposed HLR-UFS algorithm, we set the structure learning parameter to α=1. The low-rank parameter β is selected from $\left\{10^{-2},10^{8}\right\}$. Moreover, to control the sparsity of feature selection, we tune γ over $\left\{10^{-3},10^{-2},10^{-1},10^{0},10^{1},10^{2},10^{3}\right\}$. Since the local Hessian estimate relies on local normal coordinates constructed from *k*-nearest neighborhoods and PCA-based tangent-space approximation, we tune the *k*-nearest-neighbor parameter within the range of {2,3,⋯,10} for each dataset when constructing the Hessian matrix, and select the PCA subspace dimensionality accordingly. Second, for all comparison algorithms, the parameters of the different feature selection algorithms are set according to the default or recommended settings reported in their original papers. For LS, *k* = 5 is set to specify the number of neighbors. For LRSL, the structure learning parameter and the penalty weight parameter are fixed α=1 and $\beta =10^{4}$. For MRSR and RUFS, the regularized coefficient is tuned to 10. For SLNMF, the regression coefficient and the regularized term coefficient are set as $\beta =10^{-3}$ and $\gamma =10^{-2}$. For LRPFS, the penalty weight parameter and the sparse term parameter are set as 1. For RAFG, the sparse regularization parameter and the sparse level parameter are fixed as α=1, β=1, *p* = 0.7, respectively. Finally, we configure the number of selected features for the feature selector to range from 20 to 100 in increments of 10. Additionally, the maximum number of iterations is capped at 20 for all tested datasets. We evaluate the clustering performance using the *k*-means clustering algorithm with a specified number of features. Notably, the number of clusters is set to match the number of true classes for each dataset. Considering that distinct initializations can lead to diverse clustering outcomes, each experiment is conducted 30 times with random initial values. We present the mean value and standard deviation for each comparative algorithm.

### 6.4 Evaluation metrics

We evaluate the efficacy of unsupervised feature selection algorithms through the application of two widely recognized evaluation metrics: clustering accuracy (ACC) and normalized mutual information (NMI).

Here, $p_{i}$ represents the ground-truth labels provided by the dataset, while $q_{i}$ denotes the clustering labels obtained from the unsupervised feature selection algorithms. The ACC is defined as follows:


$$\begin{array}{c} \text{ACC}=\frac{\sum_{i=1}^{n}\delta (p_{i},\text{map}(q_{i}))}{n}, \end{array}$$

Here, the delta function δ(a,b) is defined such that δ(a,b)=1 if *a* = *b*, and δ(a,b)=0 otherwise. Additionally, $\text{map}(q_{i})$ denotes the mapping function that aligns the obtained clustering label $q_{i}$ with the corresponding label in the dataset.

Consider two random variables *P* and *Q*, where *P* represents the true labels and *Q* denotes the clustering results. The normalized mutual information (NMI) between *P* and *Q* is defined as follows:


$$\begin{array}{c} \text{NMI}(P,Q)=\frac{I(P;Q)}{\sqrt{H(P)H(Q)}}, \end{array}$$

Here, *I*(*P*; *Q*) denotes the mutual information between *P* and *Q*, while *H*(*P*) and *H*(*Q*) represent the entropies of *P* and *Q*, respectively. It is important to note that higher values of ACC and NMI indicate better clustering performance.

### 6.5 Result and analysis

**Fig 1:**
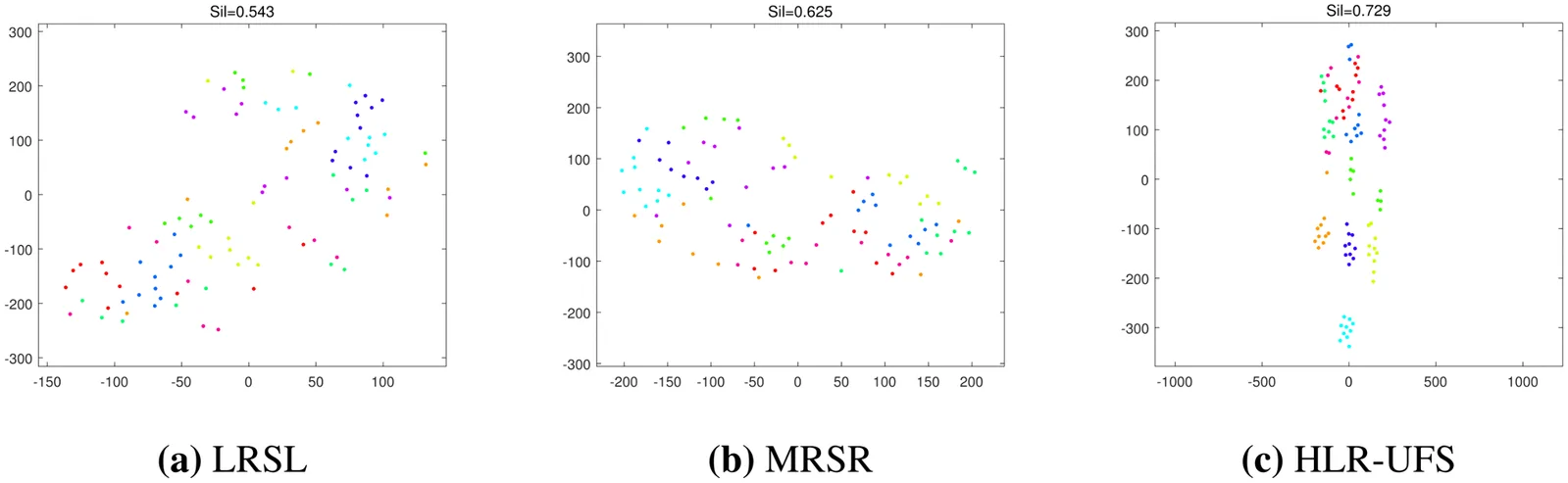
t-SNE visualization on the orlraws10P dataset.

**Fig 2:**
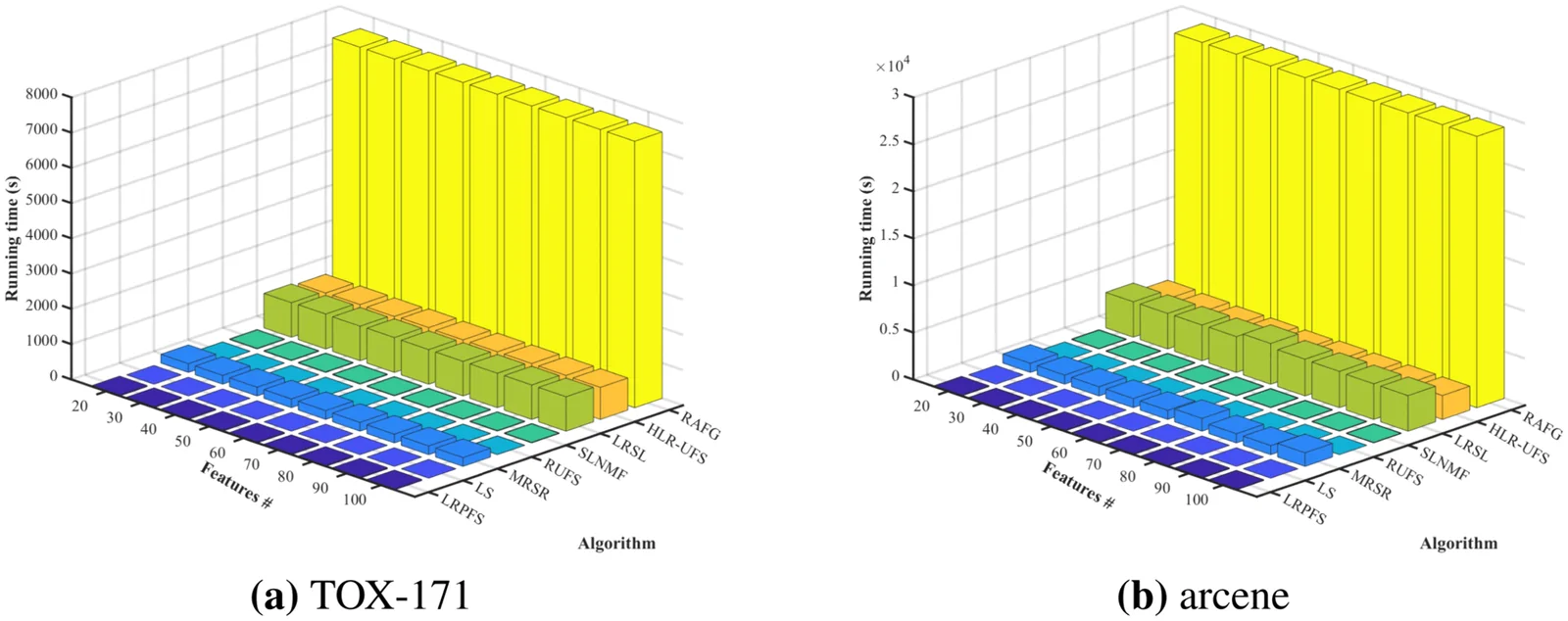
Running time comparison between HLR-UFS and all comparison algorithms on TOX-171 and arcene datasets.

In this section, we evaluate the performance of different methods on nine datasets, with a focus on ACC and NMI as the principal metrics for assessment. The ACC results are presented in Table 2, and the NMI results are detailed in Table 3. Moreover, the t-SNE visualizations and running time comparison are shown in Figs 1 and 2, respectively. These tables and figures offer a comprehensive comparison of the performance metrics and computational efficiency across various methods and datasets. From the experimental results depicted above, the following observations are made:

1) It can be seen that our method demonstrates superior performance compared to the state-of-the-art methods on nearly all tested datasets and delivers better outcomes than the Baseline, suggesting that our method can effectively exclude irrelevant or redundant features.
2) Our HLR-UFS method has better performance than the LRSL and MRSR algorithms that employ the Laplacian matrix to preserve geometrical information. For example, our method outperforms the LRSL algorithm by 6.10% in ACC on the warpAR10P dataset. This may be because compared to these two technologies, Hessian regularization possesses a richer null space than Laplacian regularization, which results in superior preservation of topological information. To further provide a direct comparison, we visualize the t-SNE embeddings of the feature subsets selected by LRSL, MRSR, and HLR-UFS on the orlraws10P dataset. As shown in Fig 1, HLR-UFS produces more compact and better-separated clusters, with higher silhouette coefficients (Sil) than the Laplacian-based methods. These results provide additional evidence that the proposed Hessian-based regularization better preserves the intrinsic higher-order geometric structure of the data.
3) HLR-UFS is slightly inferior to RAFG, the top-performing method on the arcene dataset. One possible reason is that RAFG employs a more flexible $\ell_{2,p}$-norm sparsity regularization, which may better adapt to the sparsity structure of the data and more effectively remove redundant features.
4) From Fig 2, HLR-UFS exhibits substantially shorter running time than RAFG on both TOX-171 and arcene. This is likely because RAFG combines adaptive $\ell_{1}$-norm graph learning with $\ell_{2,p}$-norm regularization and requires alternating iterative updates. HLR-UFS is also slightly faster than LRSL, possibly because LRSL optimizes a low-rank approximation model with a fixed structure-learning regularizer through alternating updates of multiple variables, which introduces additional iterative overhead.

**Table 2:** Clustering accuracy (ACC; mean ± standard deviation, %) across various algorithms on the nine tested datasets.

Datasets	LS	LRSL	RUFS	MRSR	SLNMF	LRPFS	RAFG	Baseline	HLR-UFS
warpAR10P	20.85 ± 1.58	31.36 ± 2.58	25.67 ± 2.48	26.10 ± 2.23	30.74 ± 3.52	27.51 ± 2.61	31.13 ± 2.14	27.46 ± 3.39	**37.46 ± 3.96**
arcene	25.12 ± 0.64	30.68 ± 3.79	34.00 ± 0.00	28.70 ± 3.44	25.12 ± 0.64	26.40 ± 3.36	**44.00 ± 0.00**	26.83 ± 2.96	37.30 ± 3.90
Yale_32x32	36.61 ± 1.71	33.09 ± 2.66	31.76 ± 2.30	31.62 ± 2.09	30.71 ± 2.18	30.99 ± 2.24	36.71 ± 2.80	34.30 ± 3.50	**41.92 ± 4.80**
Prostate-GE	57.84 ± 0.00	59.54 ± 0.96	60.78 ± 0.00	59.80 ± 0.00	59.22 ± 0.49	59.31 ± 0.56	53.66 ± 4.10	57.81 ± 0.18	**69.51 ± 0.70**
TOX-171	42.87 ± 2.98	42.24 ± 2.54	42.46 ± 2.40	42.53 ± 2.73	43.32 ± 1.75	41.87 ± 2.91	39.94 ± 3.46	44.21 ± 3.84	**47.52 ± 1.43**
orlraws10P	39.67 ± 3.68	37.30 ± 3.74	57.70 ± 5.80	40.07 ± 4.65	53.93 ± 6.78	37.43 ± 2.50	51.10 ± 4.77	64.07 ± 7.96	**69.67 ± 8.84**
COIL20	51.61 ± 3.38	53.36 ± 4.55	55.42 ± 4.52	47.32 ± 3.31	49.54 ± 4.04	50.21 ± 3.44	26.14 ± 2.91	52.98 ± 3.99	**56.04 ± 4.23**
JAFFE	71.92 ± 4.99	70.78 ± 7.24	69.86 ± 6.03	68.90 ± 5.93	69.78 ± 6.91	69.09 ± 7.77	66.24 ± 6.94	73.24 ± 7.61	**76.74 ± 6.04**
USPS	66.61 ± 1.94	69.63 ± 3.12	70.44 ± 3.47	68.07 ± 1.05	72.37 ± 2.42	68.69 ± 2.77	43.63 ± 2.09	70.68 ± 3.94	**76.94 ± 5.40**

Optimal results are boldfaced, second-best underlined (higher values indicate better performance).

**Table 3:** Normalized mutual information (NMI; mean ± standard deviation, %) across various algorithms on the nine tested datasets.

Datasets	LS	LRSL	RUFS	MRSR	SLNMF	LRPFS	RAFG	Baseline	HLR-UFS
warpAR10P	18.65 ± 2.26	29.72 ± 3.24	20.82 ± 2.48	21.42 ± 2.60	33.70 ± 3.41	25.45 ± 2.27	31.82 ± 1.53	28.08 ± 4.50	**42.27 ± 4.02**
arcene	1.98 ± 0.83	5.77 ± 2.76	9.08 ± 0.00	3.97 ± 2.32	1.17 ± 0.94	1.90 ± 2.46	**14.25 ± 1.19**	2.87 ± 2.57	9.08 ± 0.00
Yale_32x32	40.20 ± 1.84	40.33 ± 1.95	38.74 ± 1.97	40.56 ± 1.96	39.94 ± 1.87	39.82 ± 1.29	44.70 ± 2.11	42.07 ± 2.70	**48.41 ± 3.54**
Prostate-GE	1.79 ± 0.00	5.65 ± 0.13	5.82 ± 0.00	6.50 ± 0.00	5.27 ± 0.35	7.11 ± 0.68	2.80 ± 2.65	1.82 ± 0.00	**11.40 ± 0.75**
TOX-171	12.40 ± 1.30	15.21 ± 2.58	12.37 ± 1.31	14.54 ± 2.30	14.27 ± 1.75	14.81 ± 2.01	10.65 ± 2.39	16.73 ± 5.54	**24.62 ± 1.56**
orlraws10P	73.13 ± 2.04	46.82 ± 2.63	69.85 ± 3.18	47.09 ± 3.61	63.39 ± 5.14	45.17 ± 3.99	57.68 ± 3.33	76.15 ± 4.62	**83.42 ± 4.23**
COIL20	65.56 ± 1.27	70.14 ± 2.25	69.28 ± 1.88	62.92 ± 1.72	65.64 ± 2.09	65.70 ± 1.69	44.31 ± 1.74	72.04 ± 1.47	**72.26 ± 1.71**
JAFFE	75.47 ± 2.68	76.27 ± 4.44	72.93 ± 3.24	73.34 ± 3.36	76.47 ± 3.91	72.28 ± 3.11	71.72 ± 3.97	76.79 ± 4.56	**82.12 ± 3.51**
USPS	62.33 ± 1.47	67.07 ± 1.22	65.77 ± 1.32	64.65 ± 0.48	65.19 ± 0.96	63.24 ± 1.18	40.56 ± 0.76	66.08 ± 1.16	**68.72 ± 1.67**

Optimal results are boldfaced, second-best underlined (higher values indicate better performance).

**Fig 3:**
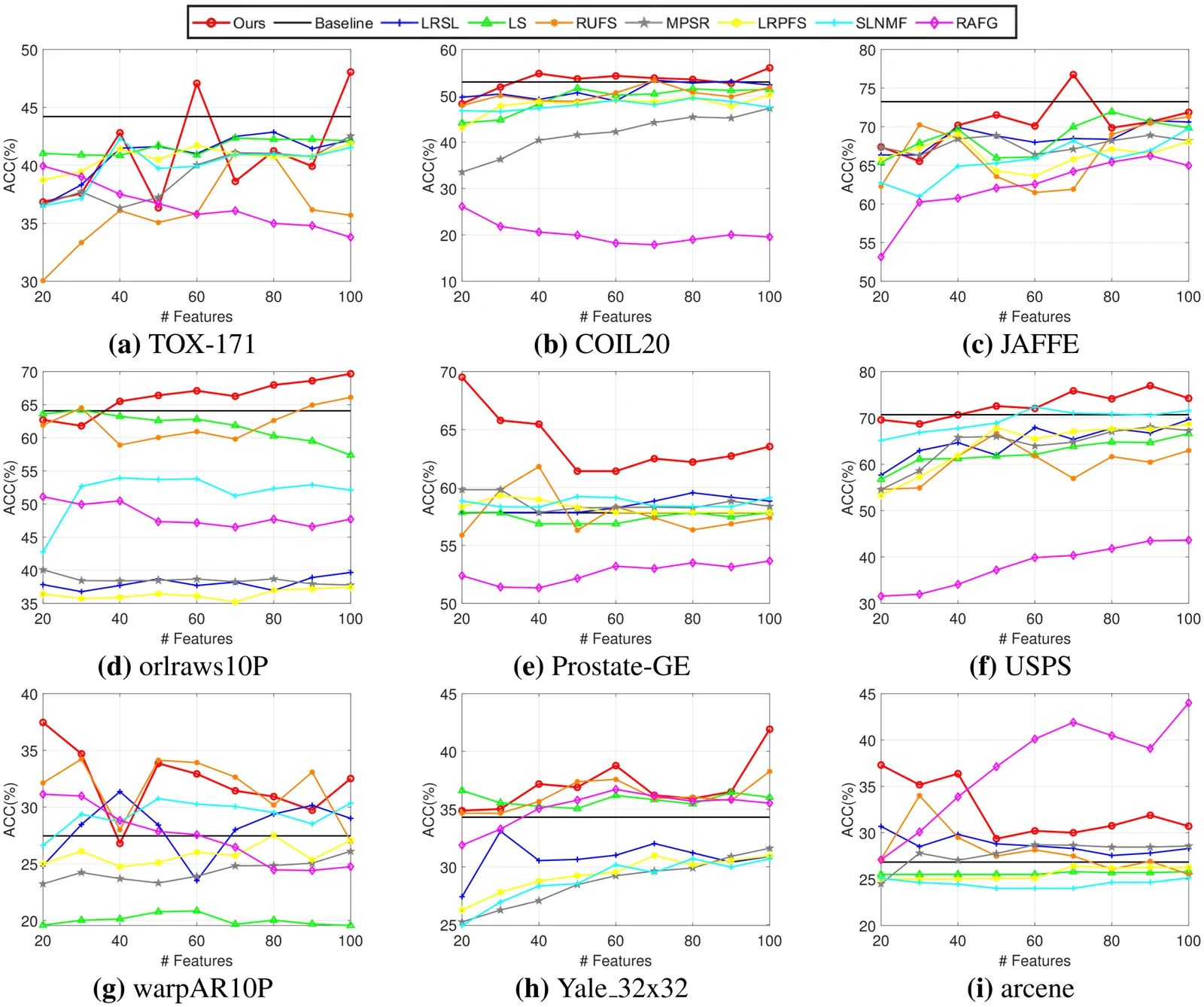
ACC values obtained by different methods using varying numbers of selected features.

**Fig 4:**
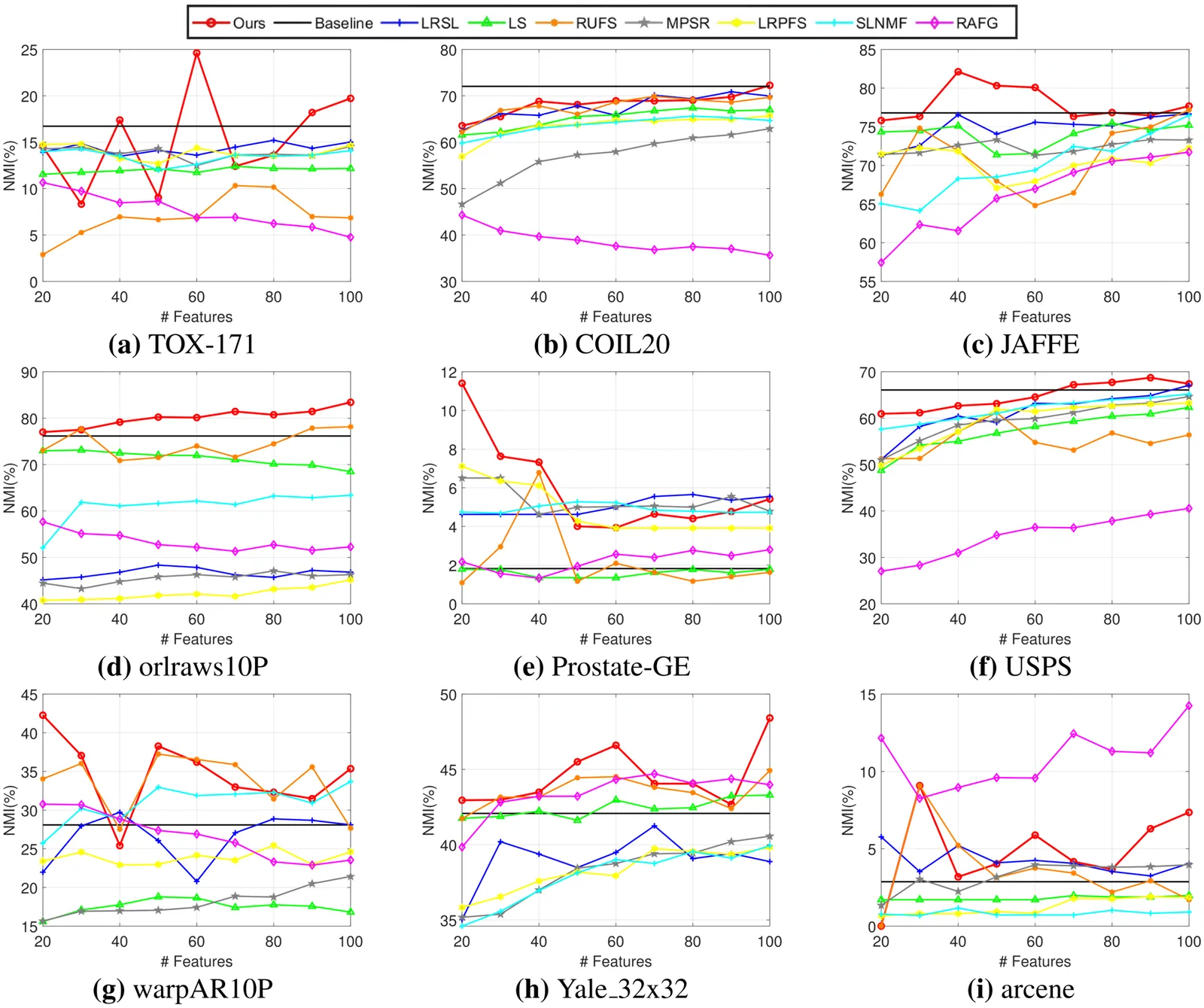
NMI values obtained by different methods using varying numbers of selected features.

Additionally, to demonstrate the impact of feature selection on clustering performance, Figs 3 and 4 provide detailed performance comparisons of all algorithms across different numbers of selected features for various datasets. On one hand, from Fig 3, the ACC results of HLR-UFS consistently outperform those of the compared algorithms across all feature numbers on the COIL20, JAFFE and Yale_32x32 datasets. On the orlraws10P and Prostate-GE datasets, when selecting a larger quantity of features, our method shows superior performance to other algorithms. On the other hand, as depicted in Fig 4, HLR-UFS also obtains the best NMI results for most feature numbers on the COIL20, JAFFE, USPS and arcene datasets. Moreover, HLR-UFS remains competitive with other methods on TOX-171 and Prostate-GE. The experimental findings consistently confirm the efficacy and advantages of the proposed HLR-UFS algorithm.

### 6.6 Parameter sensitivity

To assess the impact of the regularization parameter γ and the *k*-nearest-neighbor parameter on the clustering performance of HLR-UFS, we conduct a sensitivity analysis for each parameter individually. Experiments are conducted by fixing γ=1 and varying *k* from {2,3,...,10} or fixing *k* = 5 and varying γ from $\left\{10^{-3},10^{-2},10^{-1},10^{0},10^{1},10^{2},10^{3}\right\}$, respectively.

**Fig 5:**
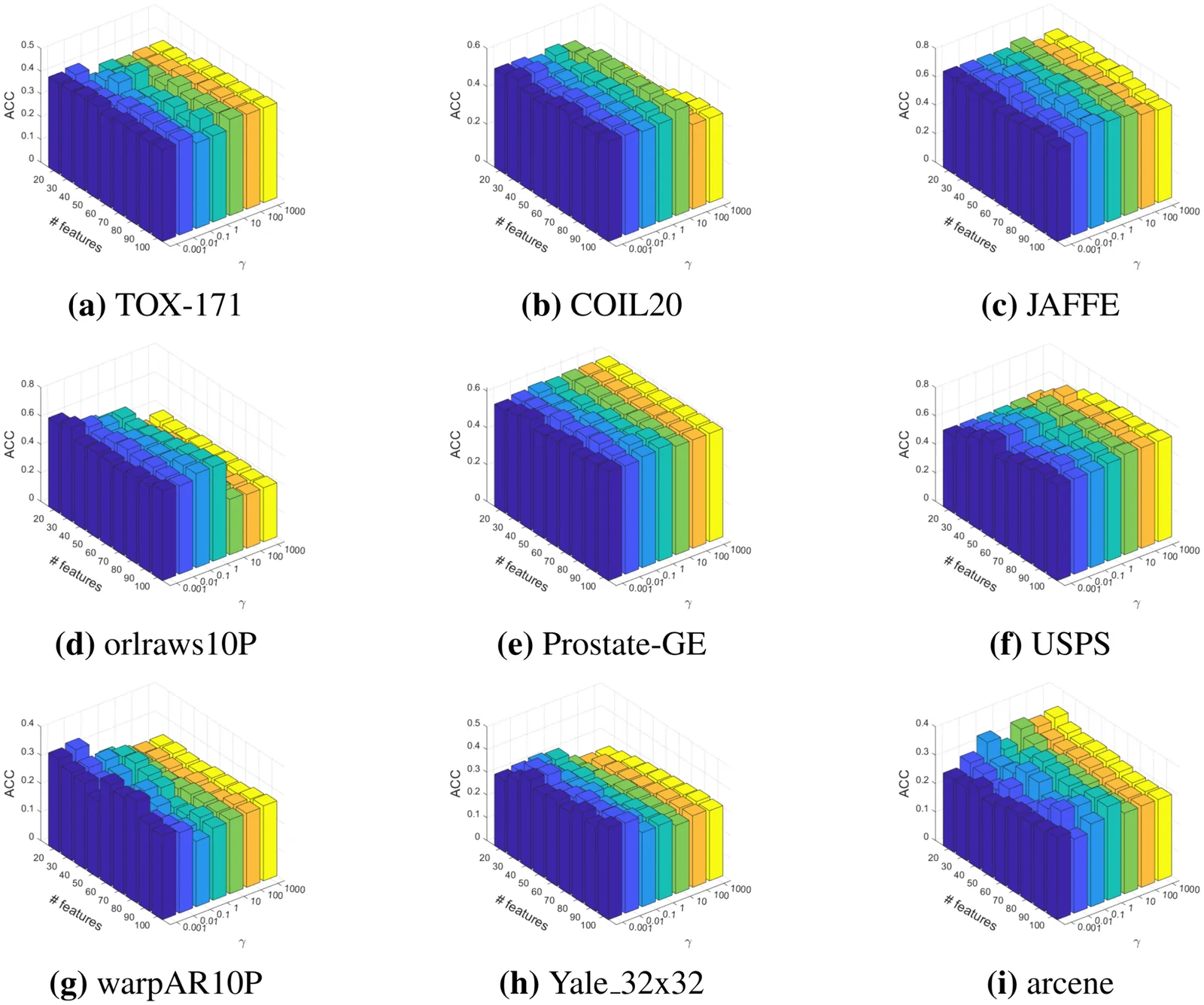
ACC of our method with respect to γ on the nine tested datasets (*k* = 5).

**Fig 6:**
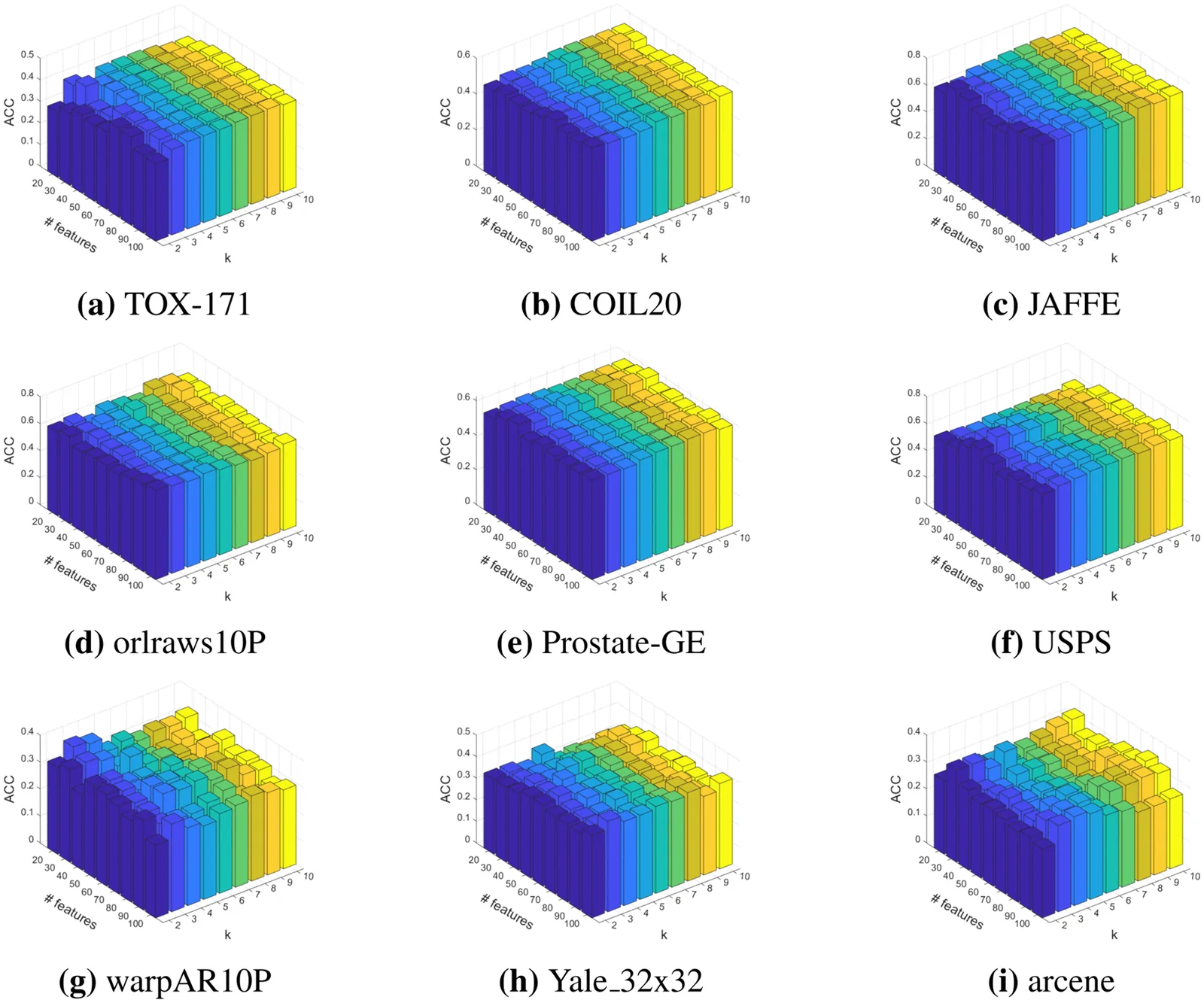
ACC of our method with respect to *k* on the nine tested datasets (γ=1).

**Fig 7:**
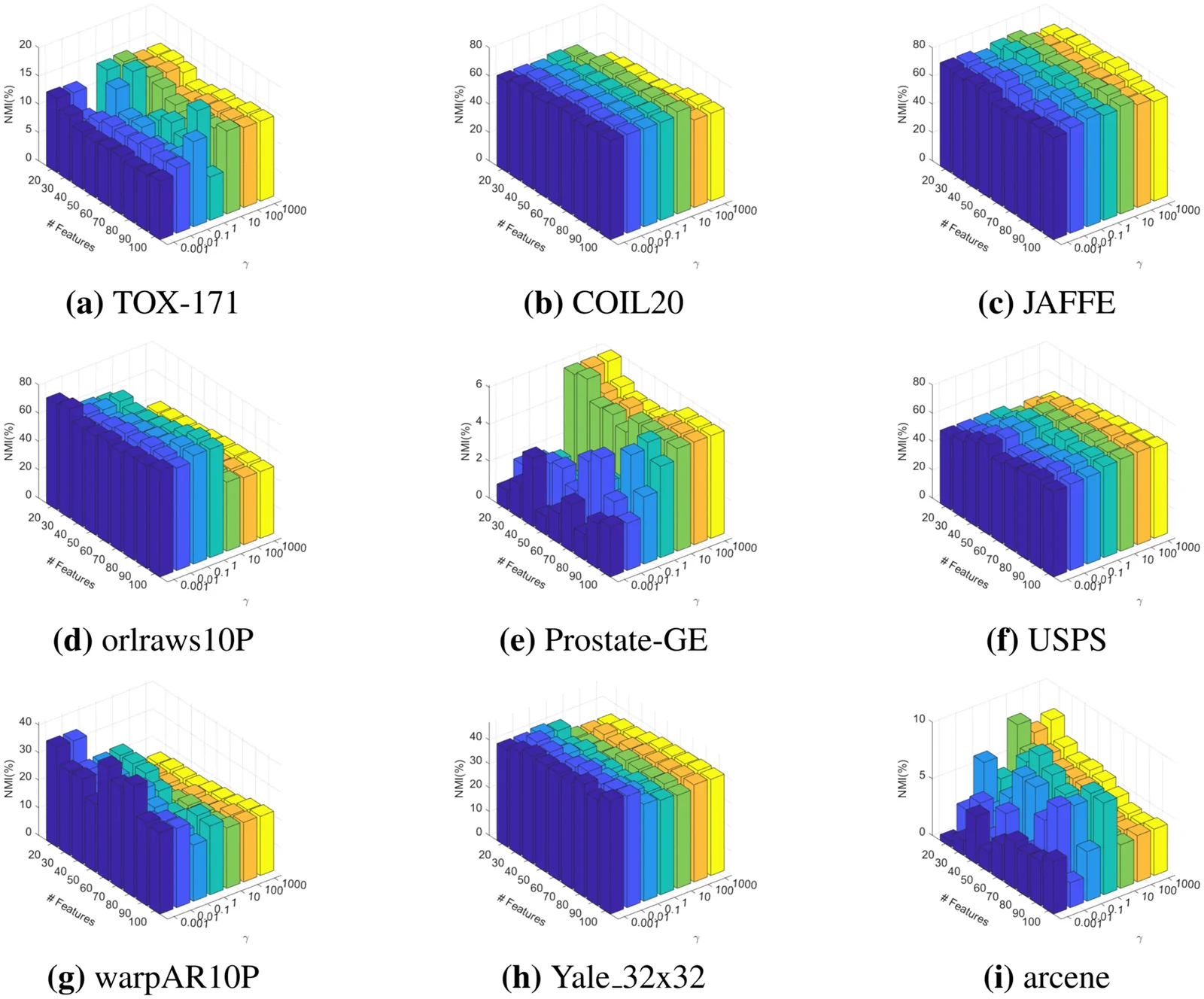
NMI of our method with respect to γ on the nine tested datasets (*k* = 5).

**Fig 8:**
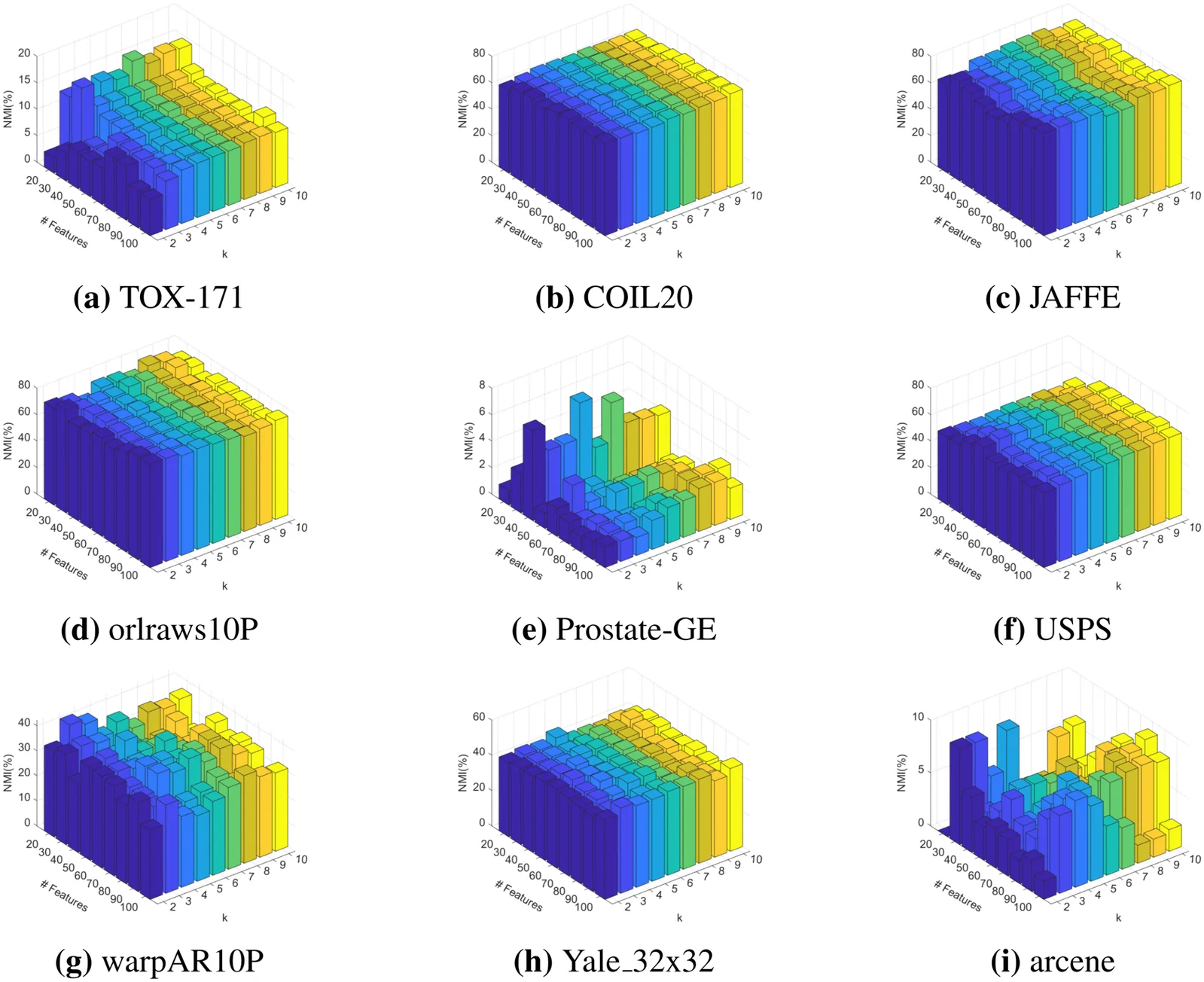
NMI of our method with respect to *k* on the nine tested datasets (γ=1).

From Fig 5, we observe that the ACC values of HLR-UFS method decrease slightly when γ is larger on the COIL20 and orlraws10P datasets and the ACC values fluctuate slightly when γ is smaller on the warpAR10P and arcene datasets. From Fig 6, the ACC values fluctuate slightly within a certain range on the warpAR10P and arcene datasets while remaining more stable on the other datasets. Notably, Fig 7 shows significant fluctuations in NMI with changes in γ on datasets like Prostate-GE, TOX-171 and arcene, indicating that the corresponding regularization terms play a vital role in the proposed model. Fig 8 indicates that the NMI exhibits pronounced fluctuations with varying *k* values on Prostate-GE, warpAR10P and arcene datasets, while it demonstrates robust performance on other datasets. Overall, these results indicate that the proposed method is generally robust to variations in both γ and *k*, as the ACC and NMI values remain relatively stable on most datasets. Meanwhile, the observed fluctuations on several datasets suggest that the parameter selection may still influence the balance of regularization terms and the reliability of Hessian-based local structure estimation.

### 6.7 Ablation study

The objective function of HLR-UFS contains three core components: Hessian regularization (controlled by α), low-rank approximation (controlled by β), and the $\ell_{2,1}$-norm sparsity term (controlled by γ). To quantify the contribution of each component, we conduct an ablation study by systematically removing one or more components and examining the resulting change in clustering performance. Specifically, we evaluate the full model and seven ablated settings on the Prostate-GE and JAFFE datasets:

- **Full Method**: the complete HLR-UFS model with all three components enabled (α,β,γ≠0).
- α=0: the Hessian regularization is removed, while the low-rank approximation and sparsity term are retained.
- β=0: the low-rank approximation is removed, while the Hessian regularization and sparsity term are retained.
- γ=0: the sparsity term is removed, while the Hessian regularization and low-rank approximation are retained.
- α=β=0: both the Hessian regularization and low-rank approximation are removed.
- α=γ=0: both the Hessian regularization and sparsity term are removed.
- β=γ=0: both the low-rank approximation and sparsity term are removed.
- α=β=γ=0: all three components are removed.

Additionally, the number of neighbors *k* is fixed at 5 in all settings. Table 4 reports ACC and NMI results.

**Table 4:** Ablation study results (ACC/NMI %) on two datasets.

Configuration	Prostate-GE	JAFFE
**Full Method**	**69.51/11.40**	**76.74/82.12**
α=0	63.17/7.10	75.09/80.29
β=0	60.78/5.93	74.19/79.77
γ=0	60.75/4.41	71.94/77.88
α=β=0	62.03/5.96	72.52/76.63
α=γ=0	60.32/3.92	70.85/76.60
β=γ=0	58.37/2.54	71.46/77.68
α=β=γ=0	57.97/2.99	72.82/77.37

Bold values indicate the best performance, and the second-best is underlined.

According to Table 4, removing any single component leads to clear performance degradation on both datasets. For example, when β=0, the ACC on Prostate-GE decreases from 69.51% to 60.78%, and the ACC on JAFFE decreases from 76.74% to 74.19%, indicating that the low-rank approximation contributes positively to the overall performance. Similar degradation can also be observed when the Hessian regularization or the sparsity term is removed.

When multiple components are removed, the performance generally deteriorates further. For instance, when α=β=0, the ACC on JAFFE drops to 72.52%, which is lower than that of the corresponding single-component removals. Removing all three components further reduces the ACC/NMI to 57.97/2.99 on Prostate-GE and 72.82/77.37 on JAFFE. Overall, these results suggest that Hessian regularization, low-rank approximation, and $\ell_{2,1}$-norm sparsity are complementary within the proposed framework, and their joint use yields the best performance among the compared settings.

### 6.8 Fixed Hessian regularization vs dynamic graph learning

Graph-based regularization techniques traditionally rely on a precomputed affinity matrix that remains fixed throughout optimization. Although effective for preserving local geometry, such a static structure may be sensitive to noise and inaccurate neighborhood estimation, since errors in the initial graph may be propagated during learning. Recent studies have therefore explored dynamic graph learning mechanisms that iteratively adapt the affinity matrix [49]. To further examine whether explicit graph updating improves robustness, we compare our fixed-Hessian framework with a representative dynamic Laplacian-based structure learning method. In the dynamic baseline, the affinity matrix is updated in each iteration via:


$$\begin{array}{c} \underset{𝐖}{\mathrm{argmin}}\sum_{i,j}\left(\|{𝐖}^{T}{𝐱}_{i}-{𝐖}^{T}{𝐱}_{j}{\|}_{2}^{2}s_{ij}+\alpha s_{ij}^{2}\right)\;\text{s.t.}\;\forall i,\;{𝐬}_{i}^{T}1=1,\;0\leq s_{ij}\leq 1. \end{array}$$ (31)

In Eq. (31), $1\in {\mathbb{R}}^{n}$ denotes an all-ones vector, ${𝐬}_{i}$ is the *i*-th row of the affinity matrix, $s_{ij}$ denotes the connectivity weight between samples *i* and *j*, and α is introduced to avoid trivial solutions. The resulting adaptive affinity matrix is denoted by $𝐒=(s_{ij})\in {\mathbb{R}}^{n\times n}$ and is iteratively refined during training.

We compare this dynamic Laplacian-based structure learning (DSL) baseline with our fixed structure learning (FSL) framework, with empirical results summarized in Tables 5 and 6. For a cleaner comparison, the sparse regularization term is removed from our formulation, yielding the FSL objective:


$$\begin{array}{cc} \underset{𝐖}{\mathrm{argmin}}\; & \|𝐗-XW{\|}_{2,1}+\alpha \mathrm{Tr}({𝐖}^{T}{𝐗}^{T}𝐇𝐗𝐖)+\beta \mathrm{Tr}\left({𝐅}^{T}𝐖{𝐖}^{T}𝐅\right)\\ \text{s.t.}\; & {𝐅}^{T}𝐅=𝐈. \end{array}$$ (32)

**Table 5:** ACC (%) comparison between the proposed fixed-Hessian framework (FSL) and a dynamic Laplacian-based structure learning baseline (DSL).

Dataset	DSL		FSL	
	Mean	Std	Mean	Std
COIL20	28.68	2.27	**54.17**	5.01
Yale 32x32	**43.15**	3.51	38.38	4.43
JAFFE	68.03	6.21	**72.85**	7.59
warpAR10P	**49.36**	4.20	37.18	4.14
USPS	38.42	0.99	**68.95**	3.78

Bold values indicate the better result.

**Table 6:** NMI (%) comparison between the proposed fixed-Hessian framework (FSL) and a dynamic Laplacian-based structure learning baseline (DSL).

Dataset	DSL		FSL	
	Mean	Std	Mean	Std
COIL20	44.14	1.69	**70.43**	2.26
Yale 32x32	**50.30**	1.96	45.89	2.49
JAFFE	75.51	2.95	**79.53**	4.58
warpAR10P	**56.79**	3.28	41.44	4.92
USPS	33.36	0.57	**61.88**	1.51

Bold values indicate the better result.

Results in Tables 5 and 6 show that the dynamic baseline does not exhibit a consistent advantage over FSL. In fact, FSL achieves better performance on three of the five datasets, indicating that the proposed fixed-Hessian framework remains competitive even without iterative graph refinement.

### 6.9 Qualitative analysis

To further investigate the robustness of HLR-UFS under structured non-Gaussian perturbations, we provide a qualitative case study on the warpAR10P dataset. We consider sunglasses as a representative form of structured perturbation and visualize the learned feature weights together with the original and reconstructed faces.

**Fig 9:**
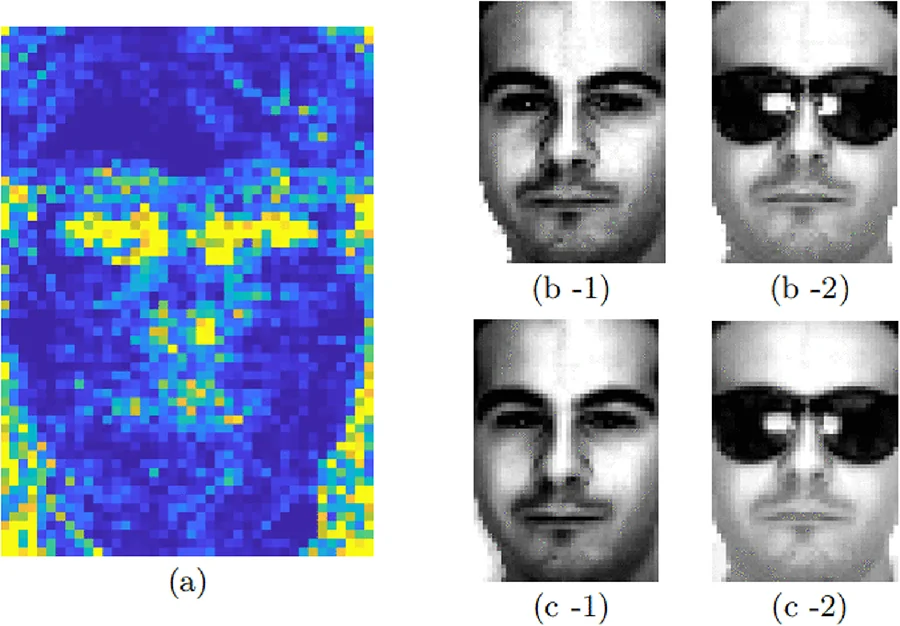
Qualitative illustration of HLR-UFS under structured sunglasses occlusion. (a) learned feature weights; (b-1) clean face; (b-2) face with sunglasses occlusion; (c-1) reconstruction of the clean face; (c-2) reconstruction of the occluded face.

As shown in Fig 9(a), the learned feature weights still emphasize several salient facial regions, especially around the eyes, nose, and mouth. Correspondingly, the reconstructed clean face in Fig 9(c-1) remains visually close to the original face in Fig 9(b-1), suggesting that HLR-UFS can preserve the main facial structure in the clean case.

We then examine the case of sunglasses occlusion. By comparing Fig 9(b-2) and Fig 9(c-2), it can be observed that the occluded region remains largely visible in the reconstructed face. This suggests that improving robustness to structured perturbations may require more adaptive and robust Hessian-based structure learning mechanisms in future work.

### 6.10 Convergence study

**Fig 10:**
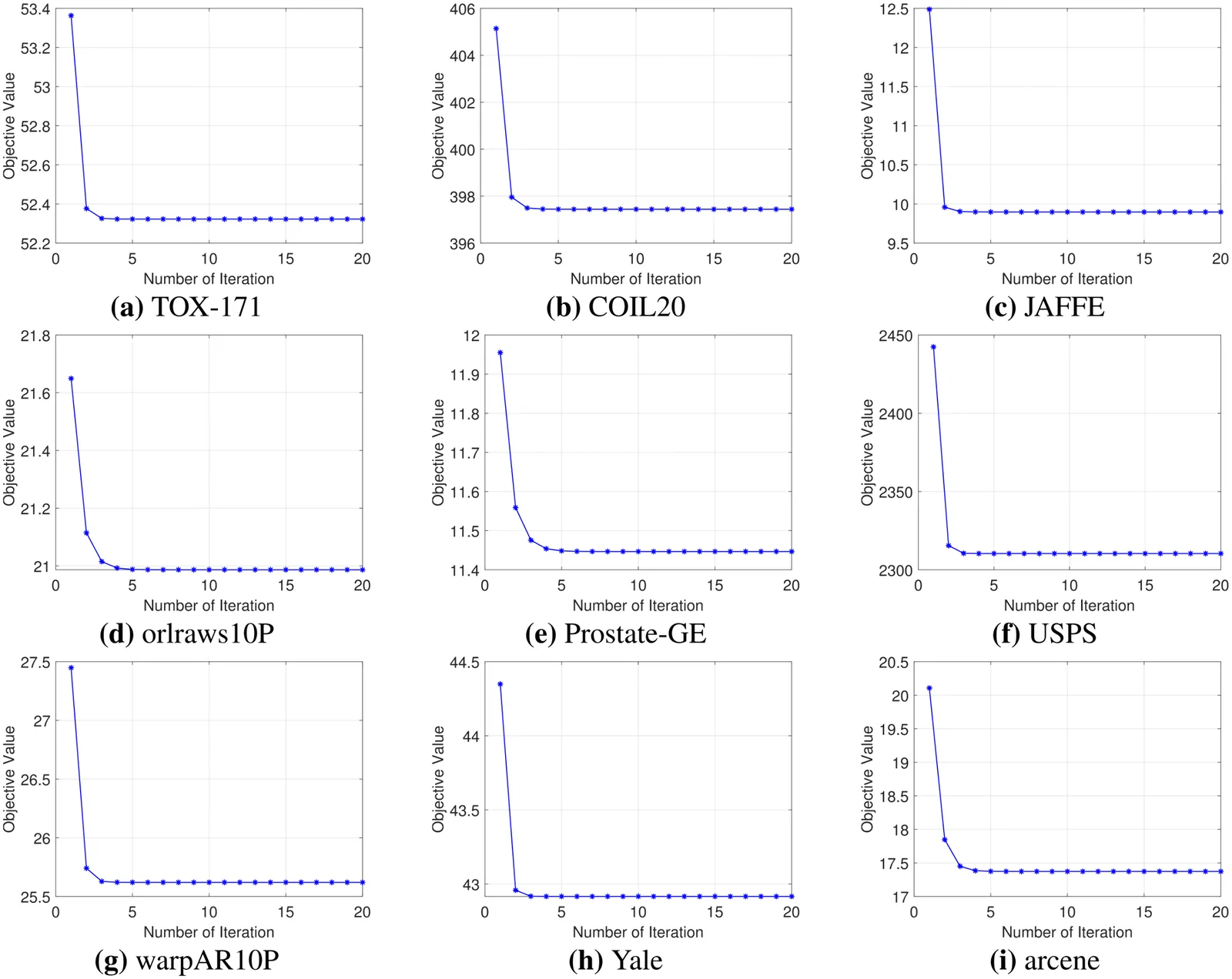
Convergence curves of HLR-UFS on the nine tested datasets.

In this subsection, we carry out an empirical analysis to examine the convergence characteristics across all the datasets that have been tested. For this purpose, we set the parameter γ to 1 and *k* to 5. Fig 10 visually depicts the convergence behavior of the HLR-UFS objective function across multiple datasets. The objective function value is observed to decrease monotonically and subsequently stabilizes following a specific number of iterations, which substantiates the accuracy and efficacy of the convergence analysis detailed in Section 5. Moreover, it is worth noting that HLR-UFS rapidly reduces the objective value and converges within five iterations on all datasets. This indicates that the optimization algorithm used for training our model demonstrates strong convergence properties.

## 7. Conclusions and future work

In this paper, we introduce an innovative framework for unsupervised feature selection, Hessian-based Unsupervised Feature Selection using Low-Rank Approximation (HLR-UFS). The HLR-UFS method addresses critical limitations in existing approaches by unifying three synergistic components: Hessian regularization to model intrinsic manifold curvature and topological connectivity, low-rank constraints to eliminate feature redundancy, and $\ell_{2,1}$-norm regularization to suppress noise and outliers. An efficient optimization algorithm based on iterative reweighted least squares is developed to solve the non-convex problem, together with convergence and complexity analysis. Finally, through comprehensive experiments conducted on nine datasets, the proposed HLR-UFS algorithm demonstrates superior performance and enhanced robustness.

Although HLR-UFS achieves competitive performance, future work will mainly focus on two aspects. First, we will investigate more adaptive and robust Hessian-based structure learning, such as dynamic neighborhood refinement and adaptive weighting, to better handle unreliable local neighborhoods, noise, and non-uniform sampling effects. Second, we will explore more scalable implementations for ultra-high-dimensional data, particularly approximate Hessian construction and distributed optimization.
